# Forensic Odontology in the Digital Era: A Narrative Review of Current Methods and Emerging Trends

**DOI:** 10.3390/diagnostics15202550

**Published:** 2025-10-10

**Authors:** Carmen Corina Radu, Timur Hogea, Cosmin Carașca, Casandra-Maria Radu

**Affiliations:** 1 Department of Forensic Medicine, George Emil Palade University of Medicine, Pharmacy, Science, and Technology of Targu Mures, 38 Gheorghe Marinescu Street, 540139 Targu Mures, Romania; carmen.radu@umfst.ro; 2 Institute of Forensic Medicine, 540141 Targu Mures, Romania; 3 Doctoral School of Medicine and Pharmacy, George Emil Palade University of Medicine, Pharmacy, Science, and Technology of Targu Mures, 540142 Targu Mures, Romania; 4 Doctoral School of Biological and Biomedical Sciences, University of Oradea, 1 University Street, 410087 Oradea, Romania; radu.casandramaria@student.uoradea.ro

**Keywords:** forensic odontology, dental identification, age estimation, bite mark analysis, human identification, artificial intelligence, intraoral scanning, machine learning

## Abstract

Background/Objectives: Forensic dental determination plays a central role in human identification, age estimation, and trauma analysis in medico-legal contexts. Traditional approaches—including clinical examination, odontometric analysis, and radiographic comparison—remain essential but are constrained by examiner subjectivity, population variability, and reduced applicability in fragmented or degraded remains. Recent advances in cone-beam computed tomography (CBCT), three-dimensional surface scanning, intraoral imaging, and artificial intelligence (AI) offer promising opportunities to enhance accuracy, reproducibility, and integration with multidisciplinary forensic evidence. The aim of this review is to synthesize conventional and emerging approaches in forensic odontology, critically evaluate their strengths and limitations, and highlight areas requiring validation. Methods: A structured literature search was performed in PubMed, Scopus, Web of Science, and Google Scholar for studies published between 2015 and 2025. Search terms combined forensic odontology, dental identification, CBCT, 3D scanning, intraoral imaging, and AI methodologies. From 108 records identified, 81 peer-reviewed articles met eligibility criteria and were included for analysis. Results: Digital methods such as CBCT, 3D scanning, and intraoral imaging demonstrated improved diagnostic consistency compared with conventional techniques. AI-driven tools—including automated age and sex estimation, bite mark analysis, and restorative pattern recognition—showed potential to enhance objectivity and efficiency, particularly in disaster victim identification. Persistent challenges include methodological heterogeneity, limited dataset diversity, ethical concerns, and issues of legal admissibility. Conclusions: Digital and AI-based approaches should complement, not replace, the expertise of forensic odontologists. Standardization, validation across diverse populations, ethical safeguards, and supportive legal frameworks are necessary to ensure global reliability and medico-legal applicability.

## 1. Introduction

Forensic odontology is a specialized and highly interdisciplinary branch of forensic science that applies dental knowledge to the service of law and justice. It encompasses the use of dental anatomy, pathology, and radiology in medico-legal contexts, thereby bridging the fields of dentistry, anthropology, and criminal investigation. The discipline plays an important role in a variety of forensic applications, including human identification, age estimation, bite mark analysis, and the assessment of orofacial trauma [1,2]. Among these, human identification is regarded as one of the most significant contributions, particularly in situations where other biometric markers such as fingerprints, facial features, or soft tissues are destroyed or unavailable [3,4]. Teeth, due to their unique morphology and exceptional durability, are often preserved even under extreme environmental conditions such as high temperatures, advanced decomposition, immersion in water, or natural disasters. This structural resilience makes them invaluable in cases of fire casualties, aviation accidents, and mass disasters, where conventional identification methods may fail [5,6].

Beyond their physical endurance, teeth also carry a wealth of individualizing characteristics. Dental restorations, orthodontic appliances, and radiographic patterns provide highly specific markers that allow for reliable comparisons between antemortem and post-mortem records [7]. As a result, forensic odontology has historically made critical contributions not only to criminal investigations and judicial proceedings but also to humanitarian missions, particularly in the context of disaster victim identification and missing persons investigations [8]. Internationally, dental experts have been mobilized in mass disasters such as earthquakes, tsunamis, and terrorist attacks, where dental records were often the primary means of establishing identity [9]. In addition to these high-profile scenarios, forensic odontology contributes on a daily basis to the resolution of civil disputes, the identification of unknown remains, and the safeguarding of human rights by helping provide closure to families of the deceased [5,10].

### 1.1. Classical Domains of Forensic Dental Determination

The primary applications of forensic odontology are well established. Dental identification involves the comparison of post-mortem findings with antemortem dental records, radiographs, or prosthetic appliances to confirm identity. Age estimation is another key application, relying on dental development in children and adolescents, as well as regressive changes such as attrition, secondary dentin deposition, or root transparency in adults. Bite mark analysis has traditionally been employed in cases of violent crime, while trauma assessment provides insights into the cause and mechanism of injuries affecting the oral and maxillofacial region. These domains illustrate the breadth of forensic dental practice and its unique contribution to both criminal justice and civil law [11,12].

### 1.2. Limitations and Controversies of Conventional Approaches

Despite their utility, traditional methods of forensic dental determination face several challenges. Morphological and odontometric analyses often depend on examiner experience and may lack reproducibility. Radiographic comparisons, while valuable, can be compromised by differences in angulation, image quality, or incomplete records [13,14]. Age estimation methods frequently show population-specific variability, limiting their generalizability across diverse groups. Moreover, the scientific reliability of bite mark analysis has been heavily debated in recent decades, with concerns regarding subjectivity, inter-examiner disagreement, and limited empirical validation [15]. These issues have prompted critical discussions about the admissibility of certain forensic dental techniques in judicial systems, particularly in the context of the Daubert or Frye standards that govern expert testimony [16].

### 1.3. Emergence of Digital Diagnostics in Forensic Odontology

In response to these challenges, technological innovation has increasingly shaped the field of forensic dentistry. Digital diagnostics—including CBCT, micro-computed tomography (micro-CT), three-dimensional surface scanning, and advanced radiographic techniques—offer non-invasive, high-resolution, and reproducible approaches to dental analysis. CBCT, in particular, has emerged as a powerful tool for visualizing dental and skeletal structures in three dimensions, facilitating age estimation, trauma evaluation, and postmortem imaging. Similarly, digital radiography and intraoral scanners have enhanced both the precision and efficiency of forensic examinations [14,15].

At the frontier of these developments lies AI and ML. These technologies are increasingly applied to automate tasks such as morphometric analysis, pattern recognition, and age or sex estimation [17]. Early studies suggest that AI models may outperform traditional methods in consistency and accuracy, though issues of dataset diversity, validation, and explainability remain unresolved. Integration with digital health records, biometric databases, and DNA analysis further enhances the multidisciplinary potential of forensic odontology, enabling more holistic and reliable identification frameworks [18]. Several AI and ML approaches have already been applied in forensic odontology. Convolutional neural networks (CNNs) have been trained on panoramic radiographs and CBCT scans for automated age estimation. Artificial neural networks (ANNs) have been used to classify morphometric measurements for sex determination, while support vector machines (SVMs) have been employed to categorize dental images into diagnostic groups. Hybrid deep learning architectures, combining CNNs with attention mechanisms, are also being investigated to improve explainability and robustness of forensic predictions.

### 1.4. Ethical, Legal, and Practical Considerations

While digital diagnostics bring unprecedented opportunities, they also raise important challenges. Standardization across methodologies is urgently needed to ensure reproducibility and comparability of results. Ethical considerations include the responsible use of personal data, the transparency of AI-driven decisions, and the protection of vulnerable populations in forensic investigations [19]. Two key ethical concerns are particularly relevant. First, transparency and explainability are essential, as courts require interpretable reasoning, yet many AI systems function as “black boxes”. Second, accountability for errors produced by AI remains unresolved, raising questions of legal responsibility in judicial contexts. To address these challenges, the implementation of standardized operating procedures (SOPs) is necessary, ensuring that AI tools are validated, their limitations documented, and their outputs interpreted under expert supervision [20].

Practical issues such as cost, training, and accessibility remain significant barriers, particularly in resource-limited settings where traditional methods still dominate. The question of legal admissibility persists as courts and legal systems evaluate whether novel digital approaches meet established standards of scientific reliability.

### 1.5. Rationale and Objectives of This Review

Given the rapid evolution of technology and the expanding body of literature on digital applications, a comprehensive review of forensic dental determination is both timely and necessary. This review aims to critically assess the current state of the field by comparing conventional methods with emerging diagnostic innovations [21]. Emphasis is placed on evaluating diagnostic accuracy, reproducibility, and legal admissibility, while also examining advances such as CBCT, intraoral scanning, three-dimensional reconstruction, and AI-driven analysis [18,22]. Moreover, this review explores the integration of dental evidence with DNA and other biometric modalities, highlighting the shift toward multidisciplinary forensic frameworks. By addressing methodological heterogeneity, ethical considerations, and barriers to implementation in resource-limited settings, the present work seeks to provide a roadmap for strengthening global forensic odontology in the era of digital diagnostics [23].

## 2. Materials and Methods

The applied methodology followed a structured approach for identifying, selecting, and critically analyzing relevant scientific literature for this narrative review.

We searched PubMed, Scopus, Web of Science, and Google Scholar databases using combinations of the terms forensic odontology, dental identification, CBCT, 3D scanning, intraoral imaging, and AI-related methodologies. Priority was given to systematic reviews, meta-analyses, and translational studies that evaluated diagnostic or therapeutic applications of forensic odontology and AI. Although not a systematic review, this approach ensures comprehensive coverage of the most relevant and up-to-date evidence, with a focus on clinical translation.

The initial search in PubMed using “forensic odontology” and “dental identification” yielded 110 results. After screening titles and abstracts, duplicate entries were removed and additional filters were applied (“AI-related methodologies”, “CBCT”, and “intraoral scanning”). Similar searches were performed in Scopus and Web of Science.

Inclusion criteria were (I) application of traditional, digital, or AI-based methods to forensic odontology (identification, age/sex estimation, bite mark analysis, trauma analysis); (II) use of radiographic, photographic, intraoral, or 3D scan datasets; (III) report of diagnostic outcomes (accuracy, reproducibility, sensitivity, specificity, predictive values); and (IV) publication as full peer-reviewed articles.

Exclusion criteria were (I) studies focused on non-forensic dental diagnostics without medico-legal relevance; (II) editorials, letters, or conference abstracts lacking methodological detail; (III) purely experimental imaging without application to forensic contexts; (IV) articles not in English; and (V) studies involving pediatric-only orthodontic imaging unless generalizable to forensic identification.

Both original research and implementation reports meeting eligibility criteria were included. Review and perspective papers were considered only for contextual background. All included studies were analyzed with respect to type and quality of imaging or dental data, forensic task definition (identification, age, sex, trauma, bite marks), AI or digital methods applied, reported performance metrics, degree of validation (internal, external, multicenter), and medico-legal considerations, including admissibility and ethical safeguards.

Given the relatively recent integration of digital technologies into forensic odontology, the search covered studies published between January 2015 and July 2025. Earlier references were cited selectively to provide historical context but were not part of the structured corpus.

**Figure 1:**
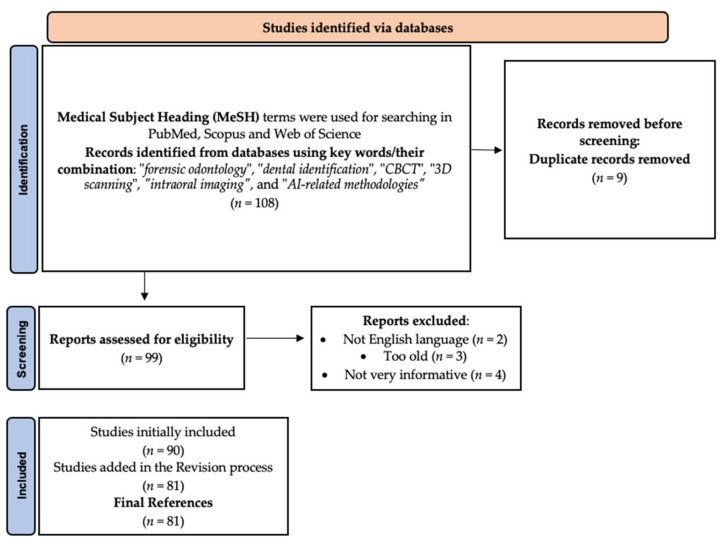
Flow diagram and selection process for the studies.

From the search, a total of 108 records were retrieved. After duplicate removal 81 articles met the eligibility criteria and were included in the final analysis. A PRISMA-style flow diagram (Figure 1) summarizes the identification and selection process.

All findings were synthesized and presented narratively, grouped into thematic sections to reflect the field’s evolution and highlight future directions.

Methodological note: This article is a narrative review, based on a bibliographic search of PubMed, Scopus, Web of Science and Google Scholar databases. The review is not systematic, and PRISMA guidelines were not applied. Instead, studies were selected for inclusion based on relevance to forensic odontology, digital diagnostics, and AI. The aim is to synthesize conventional and emerging approaches, critically evaluate their strengths and limitations, and highlight areas requiring validation.

## 3. Traditional Methods of Forensic Dental Determination

### 3.1. Human Identification Through Dental Records

One of the earliest and most widely practiced applications of forensic odontology is the comparison of postmortem dental findings with antemortem records. This method relies on the principle that no two individuals share identical dental features, and that teeth, restorations, and prosthetic devices retain distinctive characteristics throughout life. Dental charts, intraoral and panoramic radiographs, and prosthetic appliances provide valuable comparative data that can confirm or exclude identity with high levels of reliability [24]. Restorative materials, including amalgam, composite resin, glass ionomer, and porcelain crowns, are highly resistant to environmental degradation and frequently survive extreme conditions such as incineration or prolonged submersion. These features not only preserve structural integrity but also allow forensic experts to detect unique details, such as the shape and position of restorations, marginal defects, or wear patterns, which strengthen individualization [25].

This approach has become the cornerstone of disaster victim identification (DVI) efforts worldwide, being routinely employed in mass disasters such as aviation crashes, earthquakes, and terrorist attacks [26]. Interpol and other international organizations emphasize dental comparison as one of the three primary identifiers in DVI, alongside fingerprints and DNA. Nevertheless, its effectiveness depends heavily on the availability, completeness, and quality of antemortem dental records, which are often fragmented, outdated, or entirely absent in some regions. In addition, variations in record-keeping standards and access to dental care across countries may limit the global applicability of this method, underlining the need for complementary identification techniques [1,3,27].

### 3.2. Age Estimation

Age determination from dental evidence is a critical aspect of forensic odontology, with applications in both civil and criminal law. It is essential in cases involving unaccompanied minors, immigration disputes, adoption proceedings, and the identification of deceased individuals of unknown age. In children and adolescents, the stages of tooth development—including calcification, eruption patterns, and crown or root formation—are considered reliable indicators of chronological age [28]. Widely used methods include the Demirjian, Nolla, and Moorrees techniques, which assess mineralization stages of developing teeth on radiographs [29]. These techniques are non-invasive and reproducible but may show population-specific variability, necessitating the creation of regional standards [30].

In adults, where tooth development is complete, age estimation relies on regressive changes such as attrition, periodontal attachment loss, secondary dentin deposition, cementum annulations, and root transparency [31]. Gustafson’s method, for example, combines several of these regressive factors to generate an overall estimate of age. While useful, these approaches are influenced by individual variability in lifestyle, diet, and oral health, which can affect accuracy. Furthermore, examiner expertise and subjective interpretation play significant roles in reliability, highlighting the ongoing need for standardized, population-specific models that reduce bias and enhance reproducibility [32].

### 3.3. Bite Mark Analysis

Bite mark evidence has traditionally been used in cases of violent crime, particularly assaults, homicides, and sexual offenses. The principle underlying this practice is that the arrangement of dental arches and individual tooth morphology can leave characteristic impressions on skin or inanimate objects. Photographs, impressions, and overlays have been employed to compare bite marks with a suspect’s dentition. Historically, courts often admitted bite mark evidence, regarding it as highly individualistic [33].

However, in recent decades, the reliability of bite mark analysis has been increasingly questioned. Biological factors such as skin elasticity, healing, and postmortem changes can distort bite mark patterns, while technical issues such as lighting, angulation, and photographic quality further complicate interpretation. Studies have shown significant variability between examiners in analyzing the same evidence, and several wrongful convictions linked to misinterpreted bite marks have led to a reevaluation of its admissibility [34]. Professional organizations, including the American Board of Forensic Odontology, now stress the need for rigorous scientific validation and caution against overstating the evidentiary weight of bite mark analysis. While the method may still provide supportive or exclusionary information, its role as a primary identifier has diminished considerably in modern forensic practice [35].

### 3.4. Orofacial Trauma Assessment

Forensic odontology also plays a critical role in the evaluation of orofacial trauma, which includes injuries to the teeth, alveolar bone, maxillofacial structures, and surrounding soft tissues [24]. Trauma assessment provides valuable information regarding the cause, timing, and mechanism of injury, helping to distinguish between accidental and intentional harm. This is particularly relevant in cases of child abuse, domestic violence, workplace accidents, traffic collisions, and assault investigations. Patterns of injury may indicate the use of specific weapons, the direction and force of impact, or repeated episodes of trauma, thereby contributing to both criminal and civil proceedings [36].

Traditionally, these assessments have relied on clinical examination, photographic documentation, and conventional radiographs. While effective for basic diagnostic purposes, these methods often lack the ability to capture complex three-dimensional relationships or subtle fractures. For example, small alveolar fractures or intricate mandibular injuries may be overlooked on two-dimensional imaging. Such limitations have fueled the integration of more advanced diagnostic tools in recent decades, particularly three-dimensional imaging modalities such as CBCT, which provide superior visualization of hard and soft tissue injuries. Nevertheless, even conventional approaches remain valuable, particularly in resource-limited settings, underscoring the enduring relevance of traditional forensic dental trauma evaluation [37].

Traditional methods in forensic dental determination have long served as the foundation of practice, yet they increasingly coexist with emerging digital innovations.

## 4. Emerging Digital Methods in Forensic Dental Determination

The rapid development of digital technology has significantly reshaped forensic odontology, providing new tools that enhance accuracy, reproducibility, and efficiency. Unlike conventional approaches, which rely heavily on examiner expertise and two-dimensional records, digital methods offer three-dimensional visualization, automated analysis, and integration with advanced computational models. These innovations have expanded the scope of forensic dental determination and addressed many of the limitations of traditional techniques [38].

### 4.1. CBCT

CBCT has emerged as one of the most valuable imaging modalities in forensic dentistry. Unlike conventional radiographs, CBCT provides high-resolution three-dimensional reconstructions of dental and maxillofacial structures with minimal distortion. This allows for precise evaluation of anatomical features, root morphology, bone density, and trauma patterns [39]. In the context of age estimation, CBCT enables detailed visualization of secondary dentin deposition, pulp chamber size, and root transparency, offering quantitative metrics that reduce subjectivity. For disaster victim identification, CBCT imaging can be used to digitally preserve post-mortem findings, allowing comparisons to be performed remotely and repeatedly without risk of damaging physical remains. Although highly effective, CBCT requires specialized equipment and expertise, which may limit its application in low-resource forensic environments [40].

### 4.2. Micro-Computed Tomography (Micro-CT)

Micro-CT is primarily used in research but has shown great potential in forensic applications. It provides even higher resolution than CBCT, enabling microscopic evaluation of dental structures such as enamel thickness, dentinal tubules, and cementum annulations [41,42]. These features are particularly valuable for detailed age estimation and the study of regressive changes in adults. Micro-CT can also contribute to the analysis of dental trauma and material composition of restorations. However, its high cost, limited availability, and long scanning times currently restrict its widespread adoption in routine forensic practice [16].

### 4.3. Three-Dimensional Surface Scanning and Reconstruction

Three-dimensional (3D) surface scanning has revolutionized the recording and analysis of dental and craniofacial features. In forensic contexts, 3D scans of dentition, bite marks, or skeletal remains provide permanent digital records that can be stored, shared, and analyzed globally [43]. These models allow for precise morphometric analysis and virtual superimposition, enhancing the accuracy of comparisons between antemortem and post-mortem data [44]. 3D reconstruction is also increasingly applied in facial approximation and craniofacial trauma assessment, supporting both forensic investigations and courtroom presentations. The use of 3D models improves reproducibility and reduces the risk of subjective interpretation compared with manual methods [27,45].

### 4.4. Intraoral Scanners

Intraoral scanners, widely used in clinical dentistry, are now finding applications in forensic odontology [46]. These devices capture high-resolution digital impressions of the dentition, which can be used to document post-mortem dental features with great precision. Unlike conventional impressions, intraoral scanning is non-invasive, hygienic, and time-efficient. Digital records generated from scanners can be archived indefinitely and easily integrated with CBCT or other imaging data [47,48]. While intraoral scanning offers clear advantages, practical limitations include the learning curve for forensic operators and the potential difficulties of using the technology in cases involving rigor mortis or severely damaged remains [49]. Intraoral scanners have also been applied in bite mark analysis, producing high-resolution impressions that allow reproducible documentation and comparison of bite patterns. Furthermore, these devices can capture morphological distinctions of the hard palate and palatal rugae, which have been proposed as additional individualizing features in forensic identification when teeth are absent or severely damaged [50].

### 4.5. AI and ML in Forensic Odontology

AI and ML are emerging as some of the most transformative tools in forensic odontology. By leveraging computational power and advanced algorithms, AI can analyze complex dental datasets with high speed and consistency, reducing human subjectivity and enhancing reproducibility. These technologies are now being explored for several forensic applications, including age estimation, sex determination, bite mark analysis, human identification, and trauma classification [51,52,53].

#### 4.5.1. AI in Age Estimation

Dental age estimation is one of the most studied areas of AI application. Deep learning models, particularly CNNs, have been trained to assess panoramic radiographs and CBCT scans, automatically identifying developmental or regressive dental features [54]. Unlike traditional methods (e.g., Demirjian or Gustafson), which rely on predefined scoring systems, CNNs learn directly from imaging data, enabling them to detect subtle patterns beyond human perception [55]. Preliminary studies have reported improved accuracy and reduced inter-observer variability when AI systems are used, suggesting they could complement or eventually standardize age estimation in both juvenile and adult populations [56].

#### 4.5.2. AI in Sex Determination

Sex estimation using dental features has long been attempted through morphometric measurements of teeth, but these methods often suffer from population-specific variability. ML algorithms, including SVMs and ANNs, have been applied to tooth dimensions, CBCT images, and digital 3D models to improve classification accuracy. Early results suggest that automated sex prediction systems can achieve accuracy rates exceeding those of conventional morphometric methods, particularly when trained on large, diverse datasets [52,57].

Recently, generative AI and chatbot interfaces, such as ChatGPT version 5.0, have been tested experimentally as decision-support tools. These systems can assist forensic experts in applying traditional age and sex estimation methods such as Demirjian and Gustafson techniques by standardizing scoring procedures, minimizing human error, and providing rapid cross-checking against established criteria. Although promising, such applications remain preliminary and require rigorous validation before medico-legal use [58].

#### 4.5.3. AI in Bite Mark Analysis

Bite mark analysis has historically been one of the most controversial areas in forensic odontology due to issues of distortion, examiner bias, and limited reproducibility. AI offers a pathway to re-evaluate this method by applying image recognition and pattern classification algorithms to bite mark photographs or 3D surface scans [43]. CNNs, in particular, can be trained to distinguish between human and animal bite marks, detect overlapping patterns, or compare bite-marks to known dentition with greater objectivity. While research remains in its early stages, AI-driven approaches may help overcome some of the methodological weaknesses that have undermined the credibility of bite mark evidence in court [59,60].

In addition to machine learning approaches, photogrammetry has been explored for bite mark analysis. By reconstructing three-dimensional models from two-dimensional photographs, photogrammetry improves the measurement of depth, distortion, and surface topography of bite marks. When combined with AI-based classification, this technique enhances reproducibility and objectivity in forensic comparisons [61].

#### 4.5.4. AI in Human Identification

Beyond bite marks and age estimation, AI is increasingly being integrated into broader forensic identification frameworks. Algorithms can match postmortem dental images with antemortem databases, recognize restorative patterns, and even assist in reconstructing incomplete dentitions. Combined with other biometric modalities—such as facial recognition or DNA analysis—AI enhances the efficiency and reliability of disaster victim identification. Forensic case management systems are also beginning to incorporate AI modules to facilitate automated data sorting, triage, and cross-matching across large datasets [34,62].

#### 4.5.5. Challenges and Limitations of AI in Forensic Dentistry

Despite promising results, several challenges must be addressed before AI can be fully integrated into forensic odontology. The quality and diversity of datasets remain major limitations, as most current models are trained on regionally restricted populations, limiting their generalizability. Explainability and transparency are additional concerns: courts and legal systems demand clear reasoning behind forensic conclusions, yet many deep learning models operate as “black boxes,” making it difficult to justify their outputs in legal testimony [63]. Furthermore, ethical issues surrounding data privacy, consent, and algorithmic bias are particularly sensitive in forensic contexts, where decisions have direct legal consequences [64].

#### 4.5.6. Future Perspectives

The future of AI in forensic odontology lies in developing standardized, validated, and ethically robust systems. Efforts should focus on building large, open-access dental datasets that include diverse populations, ages, and pathologies. Interdisciplinary collaboration between forensic odontologists, computer scientists, and legal experts is essential to ensure that AI tools are scientifically reliable and legally admissible [65]. Hybrid approaches—where AI provides objective measurements while human experts interpret results within a broader forensic context—are likely to dominate the near future, offering a balance between automation and professional judgment [57].

The potential applications of AI in forensic odontology span from age and sex estimation to bite mark analysis and human identification. The key steps in this workflow are summarized in Table 1.

### 4.6. Integration with Multimodal Forensic Approaches

A significant advantage of digital tools lies in their ability to integrate dental evidence with other forensic modalities. CBCT and intraoral scans can be combined with DNA data, fingerprint records, or facial recognition databases to build comprehensive identification frameworks [39,66]. In mass disaster settings, digital records facilitate global collaboration, as investigators can exchange and analyze data across borders. This interdisciplinary integration enhances the reliability of identifications and reduces dependence on a single line of evidence [21].

### 4.7. Transition to Challenges and Considerations

While emerging digital methods hold tremendous promise, their widespread adoption is not without challenges. Issues such as cost, accessibility, training requirements, and legal admissibility must be addressed before these technologies can be standardized globally [63]. Moreover, the ethical use of AI and digital databases raises important questions about privacy and informed consent. These considerations underscore the importance of developing robust guidelines to ensure that technological advances in forensic odontology are applied responsibly and effectively [67].

## 5. Challenges, Limitations, and Ethical Considerations in Forensic Dental Determination

Despite remarkable progress in digital diagnostics and AI, several challenges hinder the widespread adoption and standardization of new technologies in forensic odontology.

### 5.1. Technical and Methodological Challenges

One of the most pressing issues is standardization. Current studies often employ heterogeneous methodologies, making it difficult to compare results or establish universal benchmarks [68]. Age estimation models, for example, vary widely across populations, and AI-based algorithms are often trained on regionally limited datasets that lack global representativeness. The reproducibility of results remains inconsistent, particularly in machine learning applications where “black box” algorithms may produce outputs that are difficult to interpret or validate [69].

### 5.2. Legal and Admissibility Concerns

The application of novel forensic dental methods in judicial contexts faces scrutiny under standards such as Daubert (U.S.) or Frye (common law jurisdictions) [70]. Courts require methods to demonstrate general acceptance, known error rates, peer-reviewed validation, and established protocols [71]. While conventional dental comparisons often meet these criteria, techniques like AI-driven bite mark recognition or digital reconstructions may struggle to gain admissibility due to limited validation and lack of consensus among experts [15].

### 5.3. Ethical Considerations

Ethical issues arise particularly in the context of AI and digital databases. The use of sensitive personal data, including dental records and biometric information, requires robust safeguards for privacy and informed consent [15]. Algorithmic bias is another concern: models trained on specific populations may produce less accurate or discriminatory outcomes when applied to other demographic groups. Furthermore, the explainability of AI models is essential in legal proceedings, as courts and juries require transparent reasoning behind forensic conclusions [72].

### 5.4. Practical Barriers

Even when technologies are validated, their global implementation is uneven. High costs of CBCT, micro-CT, and 3D scanning equipment limit accessibility in low-resource settings, where forensic odontologists may rely primarily on conventional radiography and manual methods [70]. Training requirements and the steep learning curve associated with AI tools and digital imaging systems further challenge adoption, particularly in regions where forensic odontology is not yet fully institutionalized [73].

### 5.5. Multidisciplinary Integration Issues

While integration of dental evidence with DNA, biometrics, and health records strengthens forensic outcomes, it also raises organizational and infrastructural challenges. Secure data sharing between institutions and across borders requires strong legal frameworks and international collaboration, which are often lacking in mass disaster or humanitarian contexts [74].

Overcoming these challenges will be essential to ensure that emerging forensic dental techniques are not only scientifically robust but also ethically responsible and legally admissible [75]. Looking ahead, future research and international cooperation will play a central role in shaping the next generation of forensic dental determination methods [62].

## 6. Discussion

Forensic odontology has progressed from traditional, examiner-dependent practices to the integration of digital technologies and AI. Classical approaches—such as dental record comparison, odontometric analysis, and radiographic imaging—remain indispensable due to their accessibility, durability of dental tissues, and established judicial acceptance. However, their reliability is often constrained by examiner subjectivity, limited reproducibility, and difficulties in cases involving degraded or incomplete remains.

Digital diagnostics, including CBCT, intraoral scanning, and three-dimensional surface reconstruction, have addressed many of these shortcomings by providing standardized, reproducible, and high-resolution datasets. These tools facilitate more objective comparisons between antemortem and post-mortem findings and enable data sharing across institutions and borders. AI further extends these capacities, with CNNs and other algorithms showing promising accuracy in age estimation, sex determination, and restorative pattern recognition. Several recent systematic reviews confirm that AI-based dental age estimation demonstrates greater reproducibility than conventional scoring systems, although results still vary across populations.

Despite these advances, important challenges remain. Validation studies are often limited in scale, with models trained on narrow demographic datasets, which reduces their global applicability. Moreover, the “black box” nature of many AI systems raises concerns about transparency and legal admissibility under standards such as Daubert and Frye. Ethical considerations, including data privacy and algorithmic bias, further complicate their routine use.

Another key barrier is implementation across diverse contexts. While CBCT, 3D imaging, and AI tools are increasingly available in high-resource forensic laboratories, their adoption is limited in low- and middle-income regions where infrastructure and funding remain inadequate. This disparity creates an uneven global landscape, in which traditional examiner-dependent methods remain dominant in much of the world. Training requirements and costs associated with advanced imaging and AI technologies also slow down their widespread integration. Ensuring equitable access will be crucial for strengthening forensic odontology as a truly global discipline.

The integration of digital and AI-based methods with other forensic modalities—including DNA analysis, facial recognition, and biometric databases—represents a promising avenue for future practice. Interdisciplinary frameworks may reduce dependence on any single identifier and improve reliability in mass disaster or humanitarian contexts. However, secure cross-border data sharing, international legal harmonization, and robust ethical guidelines are urgently needed to support such integration.

Future research should focus on large, multicenter, and demographically diverse datasets that enable the development of more generalizable AI models. Collaborative initiatives to establish open-access forensic dental databases would facilitate algorithm training, external validation, and international benchmarking. Equally important is the development of explainable AI systems, which provide interpretable outputs suitable for courtroom testimony and consistent with medico-legal standards.

Taken together, the current evidence suggests that digital and AI-based methods should be regarded as complementary rather than substitutive. Their integration with traditional techniques provides a hybrid model that strengthens forensic reliability while preserving expert oversight. The role of the forensic odontologist remains central—not only in interpreting results but also in ensuring that technological innovations are applied responsibly, ethically, and within accepted medico-legal frameworks. By combining methodological rigor, global accessibility, and interdisciplinary collaboration, forensic odontology can advance toward greater scientific credibility, legal reliability, and humanitarian impact in the digital era.

## 7. Study Limitations

The present narrative review has several limitations. As a narrative review, article selection was performed manually, which may have led to the omission of relevant studies, particularly those published in other languages. The methodological diversity across the included studies regarding datasets, algorithms, performance metrics, and forensic odontology definitions makes direct comparison difficult and hinders the formulation of standardized conclusions. No quantitative tool was used to assess the quality of the included studies, thereby reducing overall methodological rigor. Furthermore, most of the reviewed research is retrospective and originates from academic settings, which may limit applicability in routine clinical practice.

## 8. Conclusions and Future Directions

Forensic dental determination remains a vital discipline in both judicial and humanitarian contexts, providing reliable evidence for human identification, age estimation, and trauma assessment. The resilience and individuality of dental tissues ensure their enduring importance, particularly in scenarios where other biometric markers are unavailable or compromised.

The integration of digital diagnostics and AI has significantly expanded the scope of forensic odontology. CBCT, three-dimensional imaging, intraoral scanning, and AI-driven algorithms have enhanced diagnostic precision, reproducibility, and the potential for international data sharing. However, these advances also introduce new challenges, including the need for methodological standardization, population-specific validation, ethical safeguards, and legal acceptance under established admissibility frameworks.

Future progress in the field should prioritize:

**1.** **Dataset expansion and diversity**—development of large, representative, open-access dental databases to strengthen AI training and validation.

**2. International standardization**—establishment of global protocols and consensus guidelines to harmonize digital forensic dental methods.

**3. Explainable AI systems**—creation of transparent, interpretable algorithms suitable for medico-legal use and expert testimony.

**4. Equitable implementation**—promoting access to affordable digital tools and training in low-resource settings to ensure global applicability.

By combining the robustness of conventional approaches with the precision of digital innovations, forensic odontology is positioned to enhance its scientific credibility, legal reliability, and humanitarian impact in the decades ahead.

## Figures and Tables

**Table 1:** Workflow of AI Applications in Forensic Dental Determination.

Workflow Step	Description
Input Data	Dental records, radiographs, CBCT, 3D scans
AI Processing	CNNs, ANNs, SVMs
Forensic Analysis	Age estimation, sex determination, bite mark analysis
Integration	DNA, biometrics, health records
Legal & Forensic Output	Human identification, court testimony, disaster victim identification (DVI) reports

## Data Availability

No new data were created or analyzed in this study.

## References

[1] Malik S.D., Pillai J.P., Malik U. (2022). Forensic Genetics: Scope and Application from Forensic Odontology Perspective. J. Oral Maxillofac. Pathol..

[2] Kaliamoorthy S., Sethuraman V., Vijayalakshmi L. (2021). Sakthivel Sambasivam Forensic Odontology and Its Medico-Legal Applications. Indian J. Forensic Med. Toxicol..

[3] Miller R.G. (2024). Forensic Odontology in Disaster Victim Identification. J. Forensic Sci..

[4] Salazar A.M., Huerta P.A., Coliboro-Dannich V., Castro A.F., Barbaro A. (2025). Evaluation of DNA in Human Teeth-Ante-Mortem and Post-Mortem Factors Affecting Degradation and Preservation: A Literature Review. Genes.

[5] Emam A.N.M. (2024). Role of Forensic Odontology in Identification of Persons: A Review Article. Cureus.

[6] Higgins D., Austin J. (2013). Teeth as a Source of DNA for Forensic Identification of Human Remains: A Review. Sci. Justice.

[7] Ghods K., Azizi A., Jafari A., Ghods K. (2023). Application of Artificial Intelligence in Clinical Dentistry, a Comprehensive Review of Literature. J. Dent..

[8] Thammaiah S., Sheethal H., Hema K., Franklin R. (2019). Forensic Odontology as a Humanitarian Tool. J. Oral Maxillofac. Pathol..

[9] Sengupta S., Sharma V., Gupta V., Vij H., Vij R., Prabhat K. (2014). Forensic Odontology as a Victim Identification Tool in Mass Disasters: A Feasibility Study in the Indian Scenario. J. Forensic Dent. Sci..

[10] Jayakrishnan J.M., Reddy J., Vinod Kumar R.B. (2021). Role of Forensic Odontology and Anthropology in the Identification of Human Remains. J. Oral Maxillofac. Pathol..

[11] Cattaneo C. (2007). Forensic Anthropology: Developments of a Classical Discipline in the New Millennium. Forensic Sci. Int..

[12] Balachander N., Babu N.A., Jimson S., Priyadharsini C., Masthan K.M.K. (2015). Evolution of Forensic Odontology: An Overview. J. Pharm. Bioallied Sci..

[13] Rokhshad R., Nasiri F., Saberi N., Shoorgashti R., Ehsani S.S., Nasiri Z., Azadi A., Schwendicke F. (2025). Deep Learning for Age Estimation from Panoramic Radiographs: A Systematic Review and Meta-Analysis. J. Dent..

[14] Putra R.H., Doi C., Yoda N., Astuti E.R., Sasaki K. (2022). Current Applications and Development of Artificial Intelligence for Digital Dental Radiography. Dentomaxillofac. Radiol..

[15] Lacasella G.V., Signorini L., Ballini A., Bizzoca M.E., Musella G., Lo Muzio E., Cirulli N., Dimauro E., Karaboue M.A. (2025). Forensic Odontology: A Comprehensive Review of Advances and Applications in Dental Forensic Medicine. Minerva Dent. Oral Sci..

[16] Rajinikanth S.B., Rajkumar D.S.R., Rajinikanth A., Anandhapandian P.A. (2024). An Overview of Artificial Intelligence Based Automated Diagnosis in Paediatric Dentistry. Front. Oral Health.

[17] Tanveer M., Ganaie M., Beheshti I., Goel T., Ahmad N., Lai K.-T., Huang K., Zhang Y., Del Ser J., Lin C.-T. (2023). Deep Learning for Brain Age Estimation: A Systematic Review. Inf. Fusion.

[18] Sarker I.H. (2022). AI-Based Modeling: Techniques, Applications and Research Issues Towards Automation, Intelligent and Smart Systems. SN Comput. Sci..

[19] Weiner E.B., Dankwa-Mullan I., Nelson W.A., Hassanpour S. (2025). Ethical Challenges and Evolving Strategies in the Integration of Artificial Intelligence into Clinical Practice. PLoS Digit. Health.

[20] Rane J., Kaya Ö., Mallick S.K., Rane N.L. (2024). Enhancing Black-Box Models: Advances in Explainable Artificial Intelligence for Ethical Decision-Making. Future Research Opportunities for Artificial Intelligence in Industry 4.0 and 5.0.

[21] Fauziah Y.A., Darmadi E.Y., Tjandra R.R. (2024). Integrating Digital Imaging and AI in Forensic Odontology: Enhancing Identification Accuracy and Efficiency. RSF Conf. Ser. Bus. Manag. Soc. Sci..

[22] Fauziah Y., Narmada I., Darmadi E., Adityatama A., Wahjuningrum D., Yudianto A., Kurniawan A. (2025). Integration of Advanced Technology in Forensic Odontology: Revolutionizing Identification and Forensic Analysis. Res. J. Pharm. Technol..

[23] Gawali N., Shah P.P., Gowdar I.M., Bhavsar K.A., Giri D., Laddha R. (2024). The Evolution of Digital Dentistry: A Comprehensive Review. J. Pharm. Bioallied Sci..

[24] Jahagirdar P.B., Marya A., Sharma V. (2012). Role of Forensic Odontologist in Post Mortem Person Identification. Dent. Res. J. Isfahan.

[25] (2022). Corrigendum: Comparative Analysis of Commercial Colloidal Silver Products [Corrigendum] (Int J Nanomedicine., (2020), 15, (10425–10434), 10.2147/IJN.S287730). Int. J. Nanomed..

[26] Santos M.J.M.C., Zare E., McDermott P., Santos Junior G.C. (2024). Multifactorial Contributors to the Longevity of Dental Restorations: An Integrated Review of Related Factors. Dent. J..

[27] Forrest A. (2019). Forensic Odontology in DVI: Current Practice and Recent Advances. Forensic Sci. Res..

[28] Schmeling A., Dettmeyer R., Rudolf E., Vieth V., Geserick G. (2016). Forensic Age Estimation Methods, Certainty, and the Law. Dtsch. Ärzteblatt Int..

[29] Martínez Gutiérrez V.M., Ortega-Pertuz A.I. (2017). Comparison of Nolla, Demirjian and Moorrees Methods for Dental Age Calculation for Forensic Purposes. Rev. Odontológica Mex..

[30] Olze A., Reisinger W., Geserick G., Schmeling A. (2006). Age Estimation of Unaccompanied Minors. Part II. Dental Aspects. Forensic Sci. Int..

[31] Meer D., Brumit P., Schrader B., Dove S., Senn D. (2010). Root Morphology and Anatomical Patterns in Forensic Dental Identification: A Comparison of Computer-Aided Identification with Traditional Forensic Dental Identification. J. Forensic Sci..

[32] Verma M., Verma N., Sharma R., Sharma A. (2019). Dental Age Estimation Methods in Adult Dentitions: An Overview. J. Forensic Dent. Sci..

[33] Modak R., Tamgadge S., Mhapuskar A., Hebbale M., Vijayarabhavan N. (2016). Bite Mark Analysis: Chasing the Bite!. Indian J. Oral Health Res..

[34] Verma A.K., Kumar S., Bhattacharya S. (2013). Identification of a Person with the Help of Bite Mark Analysis. J. Oral Biol. Craniofacial Res..

[35] Saks M.J., Albright T., Bohan T.L., Bierer B.E., Bowers C.M., Bush M.A., Bush P.J., Casadevall A., Cole S.A., Denton M.B. (2016). Forensic Bitemark Identification: Weak Foundations, Exaggerated Claims. J. Law Biosci..

[36] Billings J., Zhan Yuen Wong N., Nicholls H., Burton P., Zosmer M., Albert I., Grey N., El-Leithy S., Murphy D., Tehrani N. (2023). Post-Incident Psychosocial Interventions after a Traumatic Incident in the Workplace: A Systematic Review of Current Research Evidence and Clinical Guidance. Eur. J. Psychotraumatol..

[37] van der Zaag P.D., Rozema R., Reininga I.H.F., van Minnen B. (2025). Standardized Clinical Examination of Maxillofacial Injury: Fracture Diagnostic Accuracy across Experience Levels. Eur. J. Trauma Emerg. Surg. Off. Publ. Eur. Trauma Soc..

[38] Gao S., Wang X., Xia Z., Zhang H., Yu J., Yang F. (2025). Artificial Intelligence in Dentistry: A Narrative Review of Diagnostic and Therapeutic Applications. Med. Sci. Monit. Int. Med. J. Exp. Clin. Res..

[39] Meto A., Halilaj G. (2025). The Integration of Cone Beam Computed Tomography, Artificial Intelligence, Augmented Reality, and Virtual Reality in Dental Diagnostics, Surgical Planning, and Education: A Narrative Review. Appl. Sci..

[40] Kapila S., Conley R.S., Harrell W.E.J. (2011). The Current Status of Cone Beam Computed Tomography Imaging in Orthodontics. Dentomaxillofac. Radiol..

[41] Swain M.V., Xue J. (2009). State of the Art of Micro-CT Applications in Dental Research. Int. J. Oral Sci..

[42] Ghavami-Lahiji M., Davalloo R.T., Tajziehchi G., Shams P. (2021). Micro-Computed Tomography in Preventive and Restorative Dental Research: A Review. Imaging Sci. Dent..

[43] Vilborn P., Bernitz H. (2022). A Systematic Review of 3D Scanners and Computer Assisted Analyzes of Bite Marks: Searching for Improved Analysis Methods during the Covid-19 Pandemic. Int. J. Leg. Med..

[44] Di Palma A., Bianchi I., Focardi M., Cioffi C., Bonetti S.S., Dalessandri D. (2024). Bitemark Analysis Comparing the Use of Digital Scans and 3D Resin Casts. J. Forensic Odontostomatol..

[45] Abraham J., Suresh K., Jacob A. (2025). Three-Dimensional Printing in Forensic Odontology: A Transformational Tool for Identification and Analysis. J. Res. Dent. Sci..

[46] Putrino A., Bruti V., Enrico M., Costantino C., Ersilia B., Gabriella G. (2020). Intraoral Scanners in Personal Identification of Corpses: Usefulness and Reliability of 3D Technologies in Modern Forensic Dentistry. Open Dent. J..

[47] Bae E.J., Woo E.J. (2022). Quantitative and Qualitative Evaluation on the Accuracy of Three Intraoral Scanners for Human Identification in Forensic Odontology. Anat. Cell Biol..

[48] Mangano F., Gandolfi A., Luongo G., Logozzo S. (2017). Intraoral Scanners in Dentistry: A Review of the Current Literature. BMC Oral Health.

[49] Santhosh Kumar S., Chacko R., Kaur A., Ibrahim G., Ye D. (2024). A Systematic Review of the Use of Intraoral Scanning for Human Identification Based on Palatal Morphology. Diagnostics.

[50] Nigliaccio S., Fontana D.A., Di Vita E., Piraino M., Messina P., Argo A., Zerbo S., Albano D., Cumbo E., Scardina G.A. (2025). Post-Mortem Animal Bite Mark Analysis Reimagined: A Pilot Study Evaluating the Use of an Intraoral Scanner and Photogrammetry for Forensic 3D Documentation. Forensic Sci..

[51] Manigandan T., Sumathy C., Elumalai M., Sathasivasubramanian S., Kannan A. (2015). Forensic Radiology in Dentistry. J. Pharm. Bioallied Sci..

[52] Khanagar S.B., Vishwanathaiah S., Naik S., A Al-Kheraif A., Devang Divakar D., Sarode S.C., Bhandi S., Patil S. (2021). Application and Performance of Artificial Intelligence Technology in Forensic Odontology—A Systematic Review. Leg. Med..

[53] Radu C.-M., Radu C.C., Zaha D.C. (2025). SmartGel OV: A Natural Origanum Vulgare-Based Adjunct for Periodontitis with Clinical and Microbiological Evaluation. Medicina.

[54] Pishghadam N., Esmaeilyfard R., Paknahad M. (2025). Explainable Deep Learning for Age and Gender Estimation in Dental CBCT Scans Using Attention Mechanisms and Multi Task Learning. Sci. Rep..

[55] Mohamed E.G., Redondo R.P.D., Koura A., EL-Mofty M.S., Kayed M. (2023). Dental Age Estimation Using Deep Learning: A Comparative Survey. Computation.

[56] Kurniawan A., Saelung M., Rizky B.N., Chusida A., Prakoeswa B.F.W.R., Nefertari G., Pradue A.F., Margaretha M.S., Alias A., Marya A. (2025). Dental Age Estimation Using a Convolutional Neural Network Algorithm on Panoramic Radiographs: A Pilot Study in Indonesia. Imaging Sci. Dent..

[57] Natarajan S., Ahmed J., Sundarraj R., Vinay V., Shetty S., Jose N.P., Chowdappa S., Carnelio S. (2025). Tooth Shape and Sex Estimation: A 3D Geometric Morphometric Landmark-Based Comparative Analysis of Artificial Neural Networks, Support Vector Machines, and Random Forest Models. 3 Biotech.

[58] Abuabara A., do Nascimento T.V.P.M., Trentini S.M., Costa Gonçalves A.M., Hueb de Menezes-Oliveira M.A., Madalena I.R., Beisel-Memmert S., Kirschneck C., Antunes L.A.A., Miranda de Araujo C. (2025). Evaluating the Accuracy of Generative Artificial Intelligence Models in Dental Age Estimation Based on the Demirjian’s Method. Front. Dent. Med..

[59] Christoloukas N., Mitsea A., Rontogianni A., Papadakis E., Angelopoulos C. (2024). Evaluation of Bitemark Analysis’s Potential Application in Forensic Identification: A Systematic Review. Diagnostics.

[60] Dhopte A., Bagde H. (2023). Smart Smile: Revolutionizing Dentistry With Artificial Intelligence. Cureus.

[61] Thali M.J., Braun M., Markwalder T.H., Brueschweiler W., Zollinger U., Malik N.J., Yen K., Dirnhofer R. (2003). Bite Mark Documentation and Analysis: The Forensic 3D/CAD Supported Photogrammetry Approach. Forensic Sci. Int..

[62] Kumar P.L., Choudhary D.J. (2023). Role of Artificial Intelligence in Technology: A Review. J. Glob. Values.

[63] Khalif Alketbi S. (2024). Emerging Technologies in Forensic DNA Analysis. Perspect. Leg. Forensic Sci..

[64] Umer F., Adnan N. (2024). Generative Artificial Intelligence: Synthetic Datasets in Dentistry. BDJ Open.

[65] Ismail M.I.B., Khan A.J., Omar N., Ahmad M.S., Tahir N.M., Yusof M.Y.P.M. (2025). Diagnostic Applications of Artificial Intelligence in Dental Care for Medically Compromised Patients: A Scoping Review. Digit. Dent. J..

[66] Shujaat S., Bornstein M.M., Price J.B., Jacobs R. (2021). Integration of Imaging Modalities in Digital Dental Workflows—Possibilities, Limitations, and Potential Future Developments. Dentomaxillofac. Radiol..

[67] Tajuddin I. (2025). Challenges of New Technology Adoption in Improving Company Growth and Competitiveness. Adv. Econ. Financ. Stud..

[68] Haugg F., Lee G., He J., Johnson J., Zapaishchykova A., Bitterman D.S., Kann B.H., Aerts H.J.W.L., Mak R.H. (2025). Imaging Biomarkers of Ageing: A Review of Artificial Intelligence-Based Approaches for Age Estimation. Lancet Health Longev..

[69] Treviño M., Arias-Carrión O. (2025). Artificial Intelligence in Systematic Reviews: Overcoming Reproducibility, Bias and Validation Challenges. Preprints.

[70] Thurzo A., Jančovičová V., Hain M., Thurzo M., Novák B., Kosnáčová H., Lehotská V., Varga I., Kováč P., Moravanský N. (2022). Human Remains Identification Using Micro-CT, Chemometric and AI Methods in Forensic Experimental Reconstruction of Dental Patterns after Concentrated Sulphuric Acid Significant Impact. Molecules.

[71] Hackman L., Nic Daeid N. (2021). Novel Scientific Methods in Court. Emerg. Top. Life Sci..

[72] Harishbhai Tilala M., Kumar Chenchala P., Choppadandi A., Kaur J., Naguri S., Saoji R., Devaguptapu B. (2024). Ethical Considerations in the Use of Artificial Intelligence and Machine Learning in Health Care: A Comprehensive Review. Cureus.

[73] Venkatesh E., Elluru S.V. (2017). Cone Beam Computed Tomography: Basics and Applications in Dentistry. J. Istanb. Univ. Fac. Dent..

[74] Nuzzolese E. (2022). Integration of Dentistry and Forensic Odontology for a Structured Identification System and Border Control. Forensic Sci. Res..

[75] Sezer B. (2025). The Role of Forensic Odontology in Modern Forensic Science: Applications, Responsibilities and Emerging Trends The Role of Forensic Odontology in Modern Forensic Science: Applications, Responsibilities and Emerging Trends Modern Adli Bilimlerde Adli D. Adli Tıp Dergisi.

